# Mitochondrial dysfunction associated with autophagy and mitophagy in cerebrospinal fluid cells of patients with delayed cerebral ischemia following subarachnoid hemorrhage

**DOI:** 10.1038/s41598-021-96092-2

**Published:** 2021-08-13

**Authors:** Dong Hyuk Youn, Youngmi Kim, Bong Jun Kim, Myeong Seon Jeong, Jooeun Lee, Jong Kook Rhim, Heung Cheol Kim, Jin Pyeong Jeon

**Affiliations:** 1 Institute of New Frontier Research, Hallym University College of Medicine, Chuncheon, Korea; 2 Chuncheon Center, Korea Basic Science Institute, Chuncheon, Korea; 3 Department of Neurosurgery, Jeju National University College of Medicine, Jeju, Korea; 4 Department of Radioilogy, Hallym University College of Medicine, Chuncheon, Korea; 5 Department of Neurosurgery, Hallym University College of Medicine, 77 Sakju-ro, Chuncheon, 24253 Republic of Korea

**Keywords:** Neuroscience, Medical research, Neurology

## Abstract

Decreased mitochondrial membrane potential in cerebrospinal fluid (CSF) was observed in patients with subarachnoid hemorrhage (SAH) accompanied by delayed cerebral ischemia (DCI). However, whether abnormal mechanisms of mitochondria are associated with the development of DCI has not been reported yet. Under cerebral ischemia, mitochondria can transfer into the extracellular space. Mitochondrial dysfunction can aggravate neurologic complications. The objective of this study was to evaluate whether mitochondrial dysfunction might be associated with autophagy and mitophagy in CSF cells to provide possible insight into DCI pathogenesis. CSF samples were collected from 56 SAH patients (DCI, n = 21; and non-DCI, n = 35). We analyzed CSF cells using autophagy and mitophagy markers (DAPK1, BNIP3L, BAX, PINK1, ULK1, and NDP52) via qRT-PCR and western blotting of proteins (BECN1, LC3, and p62). Confocal microscopy and immunogold staining were performed to demonstrate the differentially expression of markers within dysfunctional mitochondria. Significant induction of autophagic flux with accumulation of autophagic vacuoles, increased expression of BECN1, LC3-II, and p62 degradation were observed during DCI. Compared to non-DCI patients, DCI patients showed significantly increased mRNA expression levels (2^−ΔCt^) of DAPK1, BNIP3L, and PINK1, but not BAX, ULK1, or NDP52. Multivariable logistic regression analysis revealed that Hunt and Hess grade ≥ IV (*p* = 0.023), DAPK1 (*p* = 0.003), and BNIP3L (*p* = 0.039) were related to DCI. Increased mitochondrial dysfunction associated with autophagy and mitophagy could play an important role in DCI pathogenesis.

## Introduction

Subarachnoid hemorrhage (SAH) is defined as acute bleeding in the subarachnoid space due to ruptured intracranial aneurysm. Despite advances in minimally invasive surgery, the mortality rates in patients presenting with poor-grade SAH remains high: 24% for Hunt and Hess (H–H) grade 4 and 71% for grade 5^1^. Medical complications following SAH are still a challenge in neurocritical care, even if ruptured aneurysm is treated successfully. Among complications, delayed cerebral ischemia (DCI) is still a major contributor to poor neurologic outcomes of patients with SAH. The clinical syndrome of DCI is characterized by newly developed neurologic deficits with symptom fluctuation, and usually occurs between 4 and 14 days after SAH ictus^2,3^. DCI pathogenesis is known to be associated with cerebral vasospasm, cortical spreading depolarization, and microthrombosis or early brain injury (EBI). However, the mechanisms underlying DCI have yet to be elucidated. Unlike other complications including EBI, DCI is considered to be a treatable and preventable cause. Therefore, most studies on DCI are needed to provide a viewpoint different from existing ones.

Autophagy is a reparative process in which cellular homeostasis is maintained through the lysosomal degradation of dysfunctional cytoplasmic components^4^. In particular, when healthy mitochondria are maintained though the selective removal of damaged or dysfunctional mitochondria via autophagy, this is referred to as mitophagy^5,6^. Since the early 2010s, reports on mitochondrial dysfunction involved in stroke have increased^7^. Mitophagy controls the quality of the mitochondria by eliminating dysfunctional mitochondria known to contribute to the production of reactive oxygen species (ROS)^8^. Lee et al.^5^ reported a higher expression of beclin-1 (BECN1), microtubule-associated protein light chain-3 (LC3) conversion, and cathepsin-D in the frontobasal cortex, suggesting increased autophagy in neurons following SAH. Zhang et al.^9^ also showed that mitophagy was associated with oxidative stress-related neuronal death in rat SAH models induced by endovascular perforation. However, most of these studies were mainly concerned about the association of autophagy and mitophagy with EBI. EBI refers to secondary complications of SAH that usually occur within the first 72 h after ictus^10^. Accordingly, it is thought that studies on autophagy and mitophagy in DCI have hardly been done.

DCI literally means secondary brain ischemia that occurs over time rather than immediately after SAH^11^. Hayakawa et al.^7^ reported that cerebral ischemia can result in the entry of astrocytic mitochondria into adjacent neurons in mice while suppression of CD38 signaling can reduce extracellular mitochondria transfer and neurological deterioration. Under cerebral ischemic conditions, mitochondrial depolarization can lead to increased synthesis of ROS, PTEN-induced kinase 1 (PINK1), and elevated unfolded protein response^12,13^. Compared to cerebral ischemia caused by a decrease in intravascular cerebral blood, DCI has a more complex mechanism. SAH occurs in subarachnoid space which surrounds cerebral arteries. Thus, inflammation or immunological changes in cerebrospinal fluid (CSF) can affect vascular responses^11^. In addition, mitochondria can transfer into the extracellular space^14^. Therefore, analysis of CSF mitochondria may be valuable to assess the relationship between mitochondrial dysfunction and DCI.

Mitochondria in CSF show a dynamic nature following SAH. Mitochondrial membrane potential as a parameter of mitochondrial function is increased up to 3 days after ictus. It decreased thereafter^14,15^. Chou et al.^14^ reported that higher mitochondrial membrane potential in the extracellular mitochondria in CSF was associated with favorable neurologic outcomes of SAH patients. Recently, Youn et al.^15^ showed that the membrane potential was significantly decreased in mitochondria isolated from of SAH patients with DCI, but not in those without DCI. In their study, the decrease in mitochondrial membrane potential was remarkable at 5 days after ictus^15^. Moreover, mitochondria of endothelial origin shown as von Willebrand factor (vWF)-positive were increasingly detected in the CSF of DCI patient concomitant with a decreased mitochondria membrane potential. Considering the facts mentioned, we hypothesized that mitochondrial dysfunction associated with autophagy and mitophagy in CSF cells may be associated with DCI pathogenesis following SAH. However, in the case of craniotomy and clipping, there is a possibility that the analysis of mitochondria in the CSF might be affected by retraction of the brain and draining of the CSF to expose the aneurysm during surgery. Therefore, in this study, we only included SAH patients who underwent endovascular coil embolization.

## Results

### Clinical outcomes

A total of 56 SAH patients treated with endovascular coil embolization were included in the analysis (Table 1). Twenty-one (37.5%) patients experienced DCI during the follow-up. The Cohen’s kappa score for DCI diagnosis was 0.889, indicating almost perfect agreement (Supplemental Data). There was no statistical difference in clinical findings (e.g., female gender, age, hypertension, diabetes mellitus, hyperlipidemia and smoking status) between the group with DCI diagnosis and the group without DCI diagnosis. Hunt and Hess grade ≥ IV was observed in 11 (31.4%) patients without DCI and 11 (52.4%) patients with DCI. Poor outcome was observed in 20 (35.7%) patients. The number of anterior circulation aneurysms in DCI and non-DCI patients were 17 (81.0%) and 28 (80.0%), respectively.

**Table 1:** Comparison of baseline characteristics of study subjects with and without DCI.

Variables	Non-DCI (n = 35)	DCI (n = 21)	*p* value
**Clinical findings**			
Female	22 (62.9%)	17 (81.0%)	0.154
Age, years	62.7 ± 17.1	57.5 ± 10.5	0.157
Hypertension	18 (51.4%)	9 (42.9%)	0.534
Diabetes mellitus	4 (11.4%)	2 (9.5%)	0.823
Hyperlipidemia	4 (11.4%)	4 (19.0%)	0.430
Smoking	4 (11.4%)	3 (14.3%)	0.754
Hunt and Hess grade ≥ IV	11 (31.4%)	11 (52.4%)	0.120
**Laboratory findings**			
Hemoglobin	11.6 ± 1.2	12.0 ± 1.2	0.849
SaO_2_ (%)	94.9 ± 1.6	95.3 ± 1.5	0.369
Heart rate (BPM)	89.6 ± 8.0	90.5 ± 7.2	0.664
**Autophagy and mitophagy markers**^**a**^			
DAPK1	0.043 (0.021–0.086)	0.279 (0.220–0.297)	0.001
BNIP3L	0.045 (0.020–0.101)	0.134 (0.060–0.202)	0.006
PINK1	0.045 (0.012–0.063)	0.064 (0.044–0.810)	0.012
BAX	0.166 (0.095–0.223)	0.204 (0.167–0.294	0.191
ULK1	0.004 (0.001–0.008)	0.013 (0.003–0.027)	0.226
NDP52	0.155 (0.030–0.229)	0.258 (0.045–0.323)	0.155
**Radiologic findings**			
Size (mm)	5.3 ± 1.3	5.7 ± 0.9	0.225
Anterior circulation	28 (80.0%)	17 (81.0%)	0.931

*BPM* beats per minute, *DCI* delayed cerebral ischemia.

^a^mRNA levels (2^−ΔCt^) of autophagy and mitophagy markers are presented as median and interquartile range.

### Morphological changes in mitochondria of SAH patients with DCI

**Figure 1:**
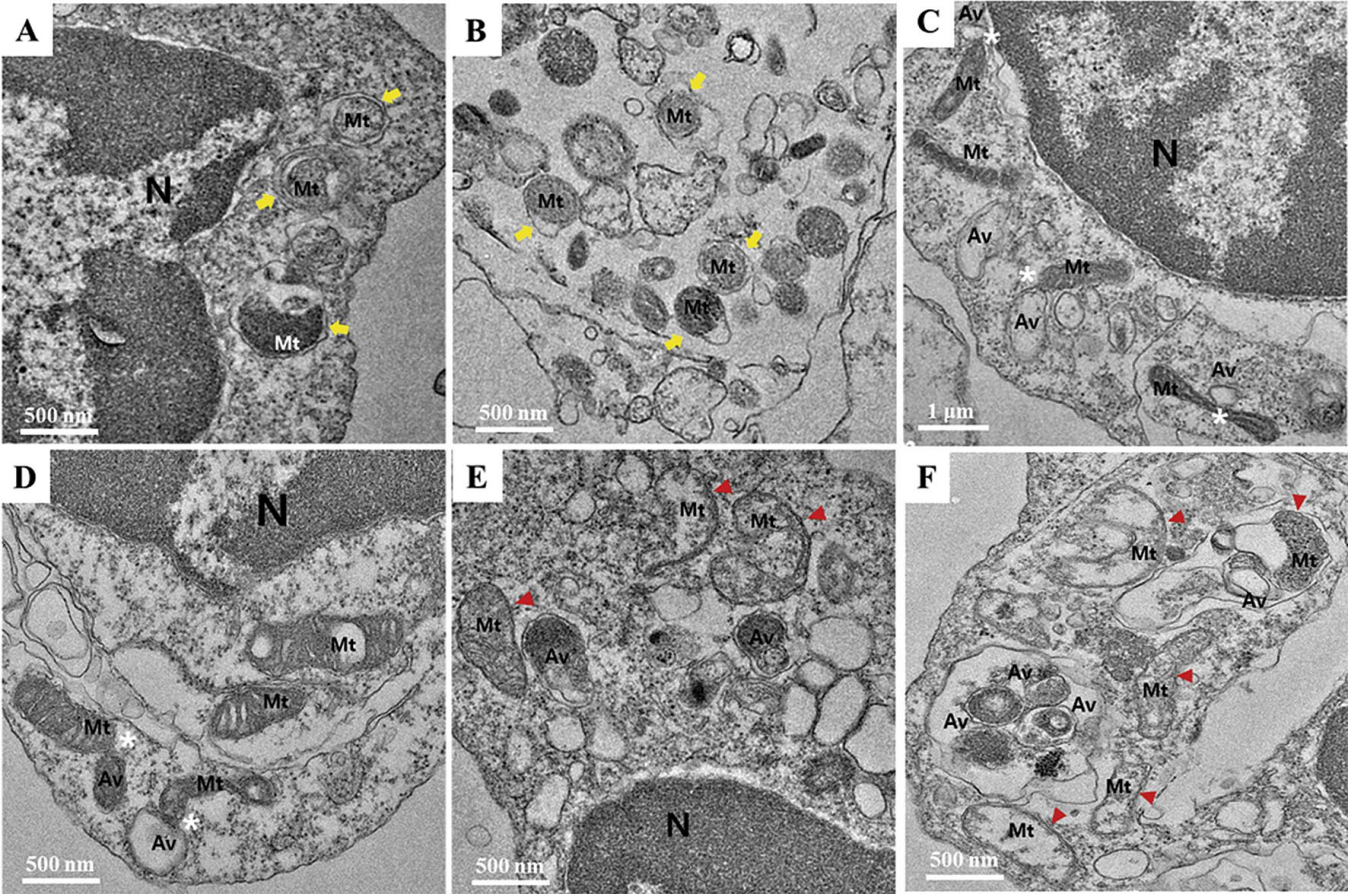
Transmission electron microscopy images of mitochondria with abnormal morphology with autophagy and mitophagy in CSF cells from SAH patients with DCI. (**A**, **B**) Autophagic vacuoles containing damaged mitochondria are indicated with yellow arrows. (**C**, **D**) Interaction between mitochondria and autophagic vacuoles is indicated by white asterisks. (**E**, **F**) Autophagic vacuoles were observed close to damaged mitochondria (red arrowhead) with matrix swelling and collapsed cristae. *Mt* mitochondria, *Av* autophagic vacuoles, *N* nuclear. Scale bar; 1 μm, 500 nm.

Transmission electron microscopy (TEM) was used to investigate the changes in subcellular ultrastructure of CSF cells obtained from SAH patients with DCI (Fig. 1). Numerous autophagic vacuoles containing mitochondria and fusion of autophagic vacuoles with abnormal mitochondria with swollen matrix and collapsed cristae accumulated in CSF cells. Additional autophagic vacuoles appeared close to the swollen mitochondria with inner mitochondrial membrane, indicating that the mitochondrial dysfunction with morphological impairment was associated with autophagic flux, and suggesting the possibility of autophagy and mitophagy in CSF cells in DCI pathogenesis.

### Increased autophagy in mitochondria of CSF cells

**Figure 2:**
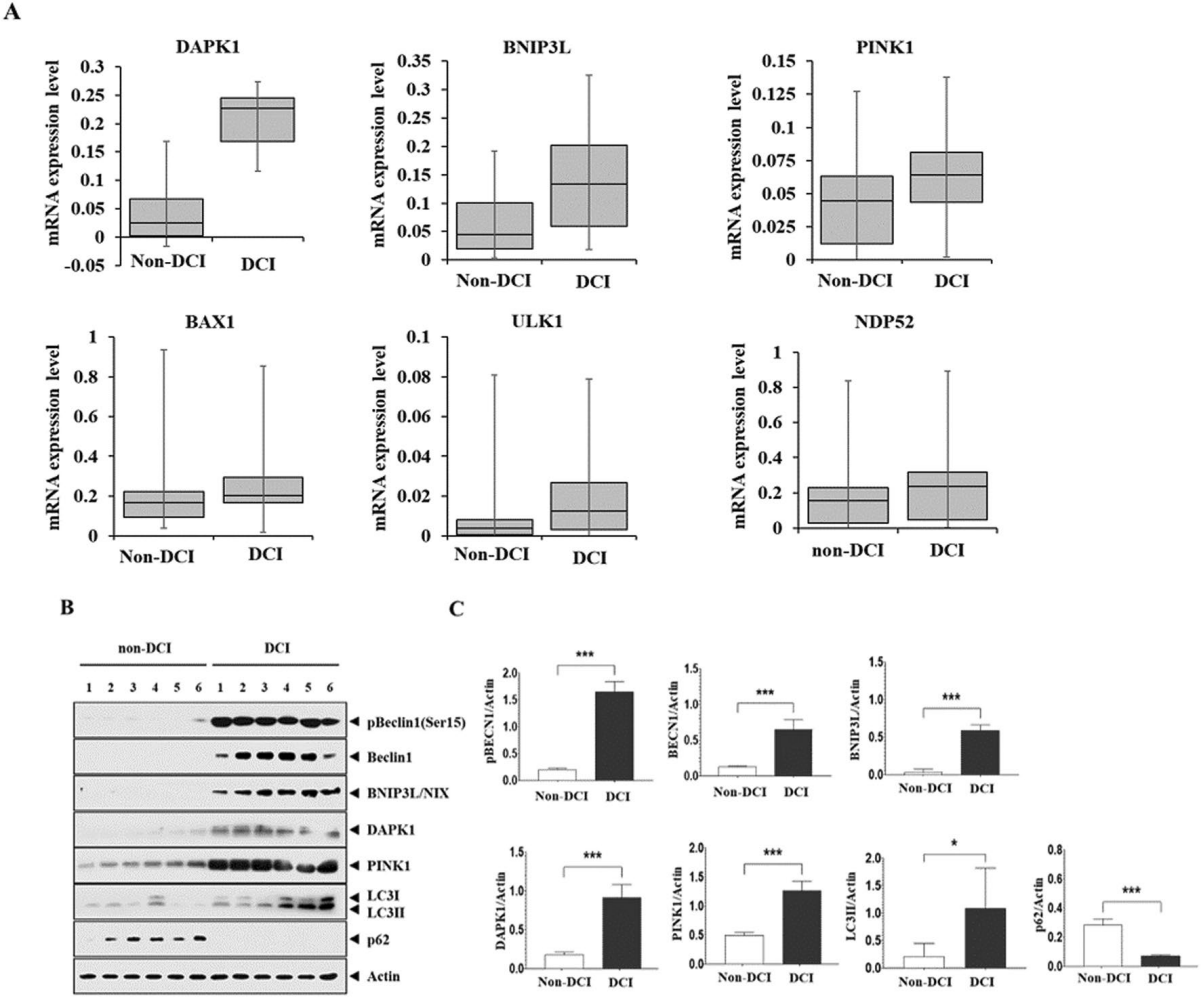
Analysis of autophagy and mitophagy markers expressed in CSF cells of SAH patients. (**A**) qRT-PCR revealed that SAH patients with DCI exhibited increased mRNA expression of DAPK1, BNIP3L, and PINK1 compared with those without DCI. Differences in mRNA expression of BAX, ULK1, and NDP52 did not reach statistical significance between the two groups. (**B**, **C**) Western blot analysis and quantification of blots using relative optical densities. Increased expression of BECN protein and phosphorylation at Ser15. LC3II expression with p62 degradation was observed in SAH patients with DCI. Actin was used as the loading control. The error bars represent SEM. *, **, and *** represent *p* value of less than of < 0.05, 0.01, and 0.001, respectively.

Quantitative real-time PCR (qRT-PCR) analysis was performed to evaluate mRNA levels of autophagy and mitophagy markers in CSF cells of SAH patients with DCI compared to those in CSF cells of SAH patients without DCI. DCI patients exhibited significantly higher expression (2^−ΔCt^) than non-DCI patients: death-associated protein kinase (DAPK)-1, 0.279 (0.220–0.297) in DCI vs. 0.043 (0.021–0.086) in non-DCI; BCL2 interacting protein 3 like (BNIP3L), 0.134 (0.060–0.202) in DCI versus 0.045 (0.020–0.101) in non-DCI, and PINK1, 0.064 (0.044–0.810) in DCI versus 0.045 (0.012–0.063) in non-DCI. Other mRNAs expressed such as Bcl-1 antagonist X (BAX), 0.204 (0.167–0.294) in DCI versus 0.166 (0.095–0.223) in non-DCI; Unc-51 like autophagy activating kinase 1 (ULK1), 0.013 (0.003–0.027) in DCI versus 0.004 (0.001–0.008) in non-DCI; and nuclear dot protein 52 (NDP52), 0.258 (0.045–0.323) in DCI versus 0.155 (0.030–0.229) in non-DCI (Table 1 and Fig. 2A). We further analyzed autophagy markers including ULK1, BECN, LC3, and p62 in CSF cells of DCI (n = 6) versus non-DCI patients (n = 6). Western blot revealed increased protein expression of BECN1 and its phosphorylation at Ser15 in DCI patients compared with non-DCI patients (Fig. 2B, C, Supplemental Figure S1, and Supplemental Table S2). In addition, increased LC3II expression and p62 degradation were also observed in DCI patients. Mitochondria dysfunction-related proteins (BNIP3L/NIX, DAPK1, and PINK1) were enhanced in the CSF cells obtained from DCI patients. Multivariable logistic regression analysis revealed that Hunt and Hess grade ≥ IV (OR = 7.515, 95% CI 1.315–42.950), DAPK1 (OR = 1.328, 95% CI 1.103–1.600) and BNIP3L (OR = 1.107, 95% CI 1.005–1.219) were related to the development of DCI following SAH (Table 2).

**Table 2:** Multivariable logistic regression analysis for the development of delayed cerebral ischemia following subarachnoid hemorrhage.

Variables	Odds ratio	95% confidence interval	*p* value
Female	0.789	0.054–11.472	0.862
Age	0.977	0.920–1.037	0.442
Hunt and Hess grade ≥ IV	7.515	1.315–42.950	0.023
DAPK1	1.328	1.103–1.600	0.003
BNIP3L	1.107	1.005–1.219	0.039
PINK1	1.291	0.994–1.676	0.055
BAX	1.634	0.999–27.986	0.282
NDP52	2.511	0.050–126.942	0.646

*BAX* Bcl-1 antagonist X, *BNIP3L* BCL2 interacting protein 3 like, *DAPK-1* death-associated protein kinase-1, *NDP52* nuclear dot protein 52, *PINK1* PTEN-induced kinase 1.

### Autophagy and mitophagy in vWF-positive CSF cells in DCI

**Figure 3:**
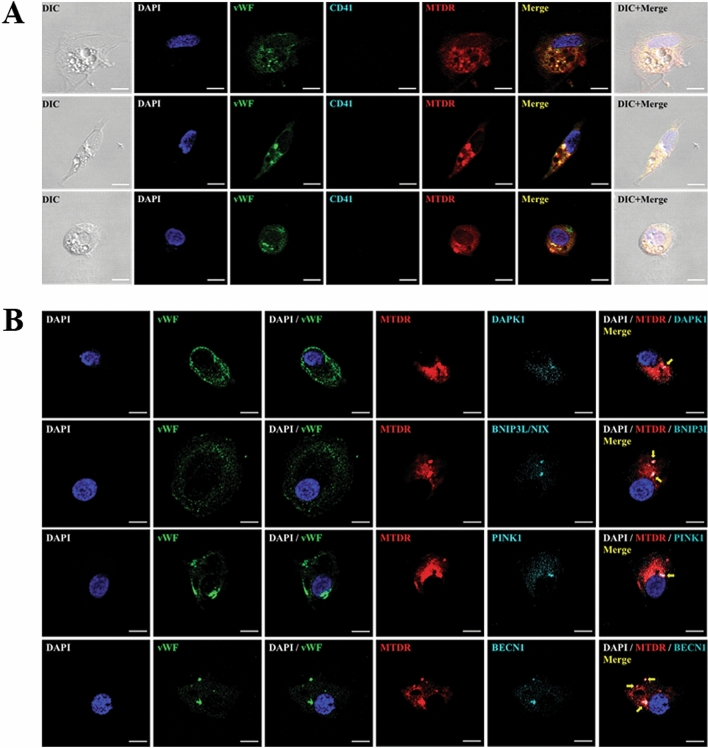
Confocal microscopy of mitochondrial dysfunction with increased autophagy and mitophagy in CSF cells from SAH patients with DCI. (**A**) CSF cells were stained with DAPI, vWF, CD41, and MTDR. Columns 1, 6, and 7 show differential interference contrast (DIC) images, merged images, and DIC with merged images, respectively. (**B**) vWF-positive CSF cells were immunostained with antibodies specific for DAPK1, BNIP3L/NIX, PINK1, and BECN1, and analyzed microscopically. White dots indicated by yellow arrows represent colocalization with autophagy and mitophagy markers in dysfunctional mitochondria under red fluorescence labeling. Scale bar, 20 μm.

To investigate whether mitochondrial dysfunction with increased autophagy and mitophagy occurred in vWF-positive CSF cells in SAH patients with DCI, multi-colored immunofluorescence staining was conducted with antibodies specific for vWF and MTDR as a marker of dysfunctional mitochondria (Fig. 3). The fluorescence signals of DAPK1, BNIP3L/NIX, PINK1, and BECN1 overlapped with that of Mito Tracker Deep Red (MTDR) in vWF-positive CSF cells. vWF-positive cells (green) were not stained with CD41 (platelet marker). The results suggested that increased mitochondrial dysfunction with autophagy and mitophagy in vWF-positive CSF cells are associated with DCI pathogenesis in SAH patients.

### Mitochondrial dysfunction concomitant with increased DAPK1 in DCI

**Figure 4:**
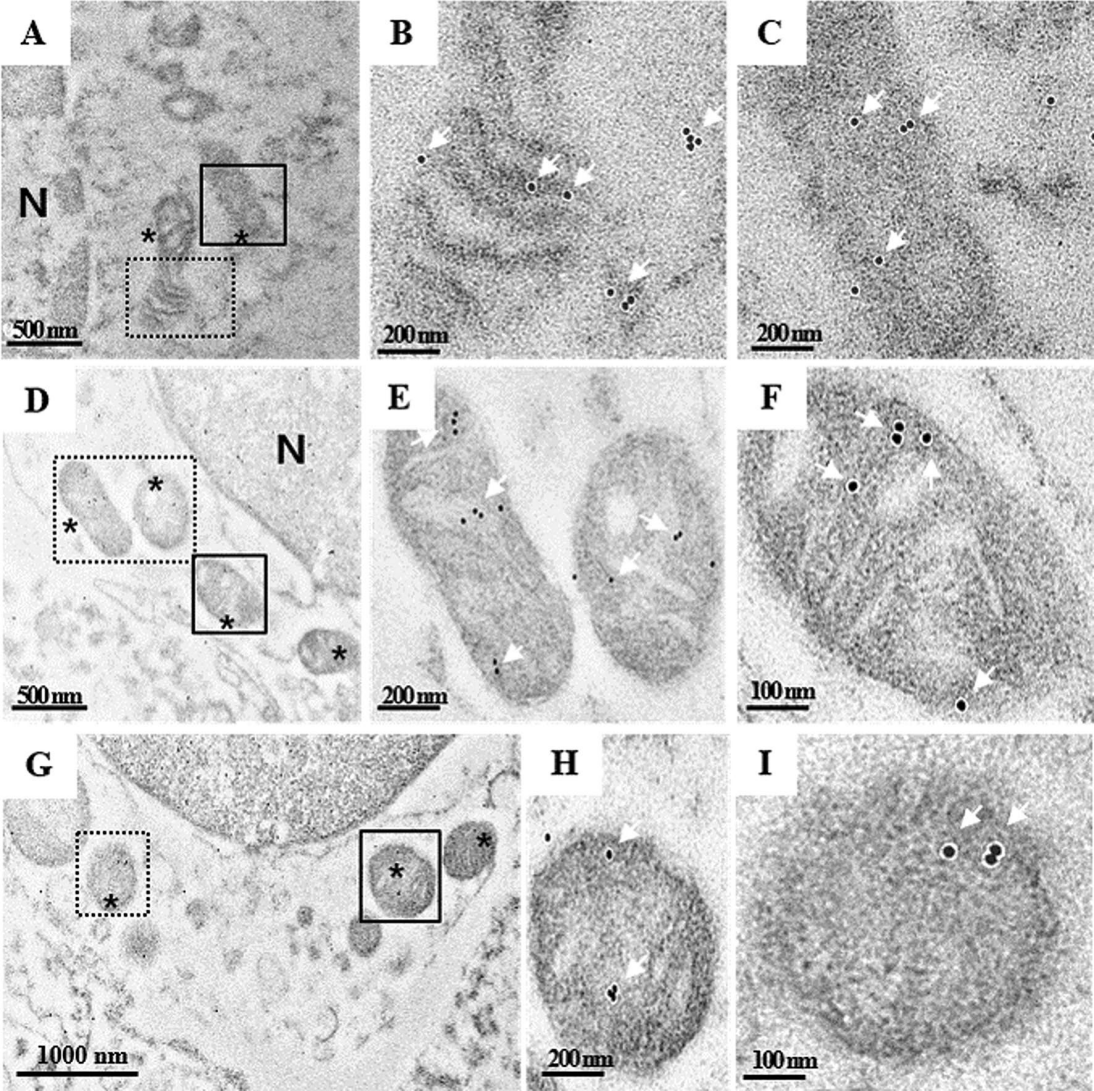
Subcellular localization of DAPK1 in CSF cells from SAH patients with DCI. Expression and localization of DAPK1 were analyzed by immunogold labeling under EM. (**A**, **D**, **G**) Damaged and swollen mitochondria with collapsed cristae are indicated with asterisks. Immunogold particles were mainly detected in mitochondria (white arrows). (**B**, **C**, **E**, **F**, **H**, **I)** Enlarged boxed areas in (**A**, **D**, **G**) respectively.

We further performed immunogold labeling with anti-DAPK1 to confirm the subcellular localization of enhanced DPAK1 in CSF cells obtained from SAH patients with DCI (Fig. 4). Interestingly, DAPK immunogold particles were mainly observed in damaged mitochondria with abnormal morphology, which is consistent with results presented in Fig. 3. Taken together, these results suggest that increased DAPK1 may play a pivotal role in mitochondrial dysfunction during the development of DCI.

## Discussion

The clinical significance of mitochondrial dysfunction has not been well studied in the DCI following SAH. In the event of cerebral ischemia, mitochondria are damaged through a series of following steps: (1) depolarization of mitochondrial membrane potential; (2) PINK2 accumulation with decreased adenosine triphosphate (ATP) reduction; (3) abnormal mitochondrial fission and fusion; and (4) broken cellular homeostasis due to increased ROS and matric calcium and subsequent cell death^12^. Autophagy and mitophagy are induced to remove damaged mitochondria after an ischemic insult. However, results did not always show only neuroprotection^12^. Baek et al.^16^ reported that attenuated autophagic signaling by carnosine, an endogenous pleiotropic peptide, was associated with improvement of mitochondrial function against ischemic brain injury. On the other hand, BNIP3L causes excessive mitophagy leading to delayed neuronal loss^17^. Chen et al.^18^ reported that -(-) Epigallocatechin-3-Gallate (EGCG) administration was beneficial for maintain autophagy flux by regulating Beclin-1 and LC3B. EGCG decreased oxyhemoglobin-induced mitochondrial dysfunction with decreased ROS production following SAH. Cao et al.^19^ also reported that melatonin lessened mitophagy-associated NLRP3 inflammasome for EBI after SAH. Nonetheless, the therapeutic role of mitophagy in the DCI is still unknown. Compared with EBI, which occurs in the brain parenchyma in the first 72 h after SAH onset, DCI usually develops 3 days thereafter and peaks from days 7 to 14. SAH literally refers to acute hemorrhage within the subarachnoid space surrounding the cerebral arteries. Free hemoglobin within the subarachnoid space triggers oxidative stress and inflammation, resulting in DCI^20^. Chou et al.^14^ initially investigated the functional relevance of mitochondria in human CSF samples following SAH. In their study, higher mitochondrial membrane potential reflected favorable functional outcomes during the 3-month follow-up. In particular, the higher percentage of mitochondria potentially originating in astrocytes was associated with a favorable outcome. In this study, we further evaluated autophagy and mitophagy markers in the CSF cells to reveal the underlying DCI pathogenesis based on mitochondrial dysfunction. Our study showed that increased mitochondrial dysfunction associated with autophagy and mitophagy in CSF cells may drive DCI pathogenesis. Among the markers, the DAPK1 expression varied most significantly between the DCI and non-DCI patients.

DAPK1 acts as a sensor of mitochondrial membrane potential in mitochondrial toxin-induced cell death^21^. Mitochondrial toxins, such as rotenone, and carbonyl cyanide m-chlorophenylhydrazone (CCCP), which is an uncoupler of mitochondrial oxidative phosphorylation induce loss of membrane potential and mitochondrial swelling leading to cell death via DAPK1 activation in neuroblastoma cells. In damaged mitochondria, the accumulation of PINK1 and LC3 on the outer membrane is accompanied by loss of membrane potential^22^. Stroke-induced neuronal cell death is related to DAPK1 signaling mediated via DAPK1-N-methyl-D-aspartate receptors (NMDARs), DAPK1-p53 and DAPK1-Tau in cerebral ischemia^23^. Tu et al.^24^ reported that DAPK1 directly binds with NR2B subunit of NMDARs in the cortex of mice, leading to aggravation of calcium reflux by enhancing the NR1/NR2B channel conductance. In their study, the genetic deletion of DPAPK1 or administration of NR2B_CT_, defined as carboxyl tail region consisting of amino acids 1292–1304, inhibited calcium reflux and protected neuronal injury against ischemic insults^23,24^. DAPK-1 was also involved in the regulation of inflammation. Activation of DAPK led to decreased T cell activation and IL-2 production^25,26^. Chuang et al.^26^ reported that DAPK was a regulator of T cell receptor (TCR)-activated NF-kappa B and T lymphocyte activation. Therefore, a further study elucidating the role of DAPK1 and T cell immunity in DCI pathogenesis in the CSF space is required.

In this study, we further evaluated the role of confocal microscopy to confirm the differential expression of autophagy and mitophagy markers in vWF positive-CSF cells of SAH patients with DCI^14^. The findings demonstrated colocalization with DAPK1, BNIP3L, PINK1, and BECN1-positive autophagosomes in mitochondria, suggesting mitochondrial dysfunction with autophagy and mitophagy in DCI pathogenesis. Endothelial dysfunction is related to cerebrovascular disease. Nitric oxide and endothelin released from endothelium regulates vascular function^27^. Compared with healthy controls, SAH patients exhibit elevated synthesis of endothelial microparticles^28^. In addition, increased levels of CSF and plasma endothelin were observed in SAH patients with vasospasm^27^. In DCI patients, a higher percentage of vWF-positive mitochondria was observed compared with non-DCI patients^15^. Accordingly, future studies should focus on the alleviation of the mitochondrial dysfunction in CSF cells of endothelial origin in DCI patients.

This study has some limitations. First, we enrolled SAH patients treated with coil embolization, but not surgical clipping. The penetration of laminar terminalis or brain retraction injury during the clipping can contaminate the CSF component, and therefore, bias the results of CSF mitochondria. Therefore, we excluded them from our analysis. We did not include a control group either. Thus, a follow-up study including SAH patients who underwent clipping and a control group should be performed in the future. Second, no medical treatment or additional chemical angioplasty for DCI severity was considered in the interpretation of results. In general, suppression of autophagy contributes to increased cell death, but can lead to cytotoxicity under some circumstances^29^. Mitochondrial dysfunction is likely to vary depending on the initial SAH severity and thus may be associated with the patient’s outcome. Although there was no statistical significance of autophagy and mitophagy markers according to the SAH severity and patient’s outcome (Supplemental Table S3 and S4), additional analysis should be performed using a large number of the SAH patients. Nevertheless, our study represents the first investigation of mitochondrial dysfunction associated with autophagy and mitophagy in CSF cells derived from SAH patients with DCI.

## Conclusions

Increased mitochondrial dysfunction with autophagy and mitophagy could play an important role in DCI pathogenesis of patients with SAH.

## Materials and methods

### Study population

The derivation cohort was derived from the regional stroke database between March 2016 and May 2020. We selected SAH patients from this database based on the following inclusion criteria: (1) adult patients > 18 years old; (2) SAH due to ruptured aneurysm; (3) dense localized clot and/or vertical layer of blood greater than 1 mm in thickness on computed tomography (CT); and (4) SAH patients who were treated with endovascular coil embolization. The exclusion criteria were: (1) non-aneurysmal SAH such as trauma, infection or perimesencephalic SAH, (2) patients treated with surgical clipping and (3) previous history of central nervous system disorder or mitochondrial diseases^30^.

TEM was used to detect the autophagic vacuoles and morphological changes of mitochondria in CSF cells derived from SAH patients with DCI. We evaluated and compared the autophagy and mitophagy biomarkers in CSF cells of SAH patients with and without DCI using qRT-PCR. The markers included DAPK-1, BNIP3L, BAX, PINK1, ULK1 and NDP52. We further evaluated the expression of autophagy executor gene of BECN1 and autophagy adaptor protein of p62^31,32^. Confocal microscopy was used to identify colocalization of differentially expressed genes in vWF-positive CSF cells, which represented endothelial cell origin, and were increased in SAH patients with DCI^15^.

In our study, the diagnosis of DCI wad performed through the following criteria: (1) new developed neurological changes such as motor weakness, dysphasia, and sensory change; (2) decrease consciousness by more than 2 points via the Glasgow Coma Scale score or National Institutes of Health stroke scale; (3) fluctuation of symptoms lasing more than 1 h; (4) cerebral infarction identified on CT or MRI, but not complications related to the endovascular coil embolization; (5) concomitant severe cerebral vasospasm with narrowing more than 50% compared to the initial radiological tests; and (6) excluding other causes that may neurological changes such as re-bleeding, hydrocephalus, seizures or electrolyte imbalances^2,33^. When DCI was suspected, treatment to increase blood pressure was given first. Blood pressure was targeted with the mean systolic blood pressure 20 mm Hg above the baseline, up to 200 mm Hg while looking at the clinical response. Intra-arterial chemical angioplasty using nimodipine was performed when DCI-related clinical symptoms worsened or severe vasospasm was suspected based on a TCD greater than 200 cm/s in the middle cerebral artery or 85 cm/s in the basilar^34^, despite treatment to induce hypertension. The procedure was carried out once or twice a day from 1 to 7 days after identification of DCI. Poor outcome was defined when a modified Rankin scale (mRS) score was three or more at 3-month follow-up.

After coil embolization, continuous lumbar drainage of CSF was maintained in the neurointensive care unit in every SAH patient for 1 week after ictus in our institution. Because difference in mitochondrial membrane potential of the CSF cells was most prominent on day 5 post ictus in SAH patients with and without DCI^15^, we analyzed the CSF samples obtained from days 5 to 7.

Clinical, laboratory, and radiological information was reviewed by the two investigator independently. Disagreements were resolved by the third investigator. The protocol of DCI diagnosis among reviewers are presented in detail in the Supplemental Data. Sample collection and study design were performed according to the principles of the Declaration of Helsinki and were approved by the Institutional Review Board of the Chuncheon Sacred Heart Hospital (No. 2017–9, 2018–6, and 2019–6). All methods were performed in accordance with the relevant guidelines and regulations in manuscript. Informed consent was received from the patients or their relatives.

### Transmission electron microscopy

Previously, SAH patients with DCI showed depolarization of mitochondrial membrane potential, which triggers alteration in mitochondrial function and morphologies^15^. In the present study, TEM was used to investigate the changes in subcellular ultrastructure of CSF cells in SAH patients with DCI. CSF samples were centrifuged at 4000 rpm for 10 min, and the pellets were analyzed by electron microscopy^15^. The pellets were fixed overnight in 2% glutaraldehyde in cacodylate buffer (0.1 M sodium cacodylate, 2 mM MgCl2) at 4 ℃. After washing three times with cacodylate buffer at 4 ℃, the samples were post-fixed in 2% osmium tetroxide for 1 h at 4 ℃. The samples were rinsed with deionized water and dehydrated through a gradient series of ethanol, ranging from 50 to 100% ethanol, 20 min each step. The samples were incubated with progressively concentrated propylene oxide dissolved in ethanol followed by infiltration with increasing concentration of Eponate 812 resin. Samples were baked in a 65 °C oven overnight and sectioned using an Ultra microtome. Sections were viewed with a Field Emission TEM unit (JEM-2100F, JEOL) at the Korean Basic Science Institute, Chuncheon, South Korea^15^.

### Western blot analysis

CSF cells obtained from SAH patients were lysed with RIPA buffer (50 mM Tris-base, 10 mM EDTA, 150 nM NaCl, 0.1% SDS, 1% Triton X-100, 1% sodium deoxycholate, 1 mM PMSF). Protein lysates of the supernatant were quantified using the BCA protein assay kit (Thermo Scientific, USA). Equal amounts of protein were separated on 10% SDS–polyacrylamide gel electrophoresis (SDS-PAGE) and transferred to PVDF membranes (Bio-Rad, USA). After blocking the membranes with 2% BSA in TBS-T (Tris-buffered saline including 0.1% Tween-20) for 1 h, the membranes were incubated with the primary antibodies overnight at 4 ℃. After extensive washing, the membranes were incubated with HRP-conjugated secondary antibodies, and developed using an enhanced chemiluminescence (ECL) kit (Thermo Scientific, USA). Antibodies used in this study were: BECN1 (#3495, Cell Signaling Technology, USA, dilution 1:1000), p62 (sc-48402, Santa Cruz Biotechnology, USA, dilution 1:1000), LC3 (#3868, Cell Signaling Technology, USA, dilution 1:1000), pBECN1Ser15 (#84,966, Cell Signaling Technology, USA, dilution 1:1000), DAPK (PA5-14,044, Invitrogen, USA, dilution 1:1000), PINK1 (ab23707, Abcam, UK, dilution 1;1000), BNIP3L/NIX (ab8399, Abcam, UK, dilution 1:1000), and actin (sc-47778, Santa Cruz Biotechnology, USA, dilution 1:1000).

### Immunofluorescence staining

To evaluate the colocalization of autophagy and mitophagy markers with dysfunctional mitochondria in vWF-positive CSF cells, multi-color immunofluorescence staining was performed via confocal microscopy. CSF cells were fixed with paraformaldehyde (4% w/v) and then washed with PBS. After blocking with 2.5% blocking solution (Vector, USA) for 1 h, the cells were incubated with antibodies specific to DAPK1 (PA5-14,044, Invitrogen, USA, dilution 1:100), PINK1 (ab23707, Abcam, UK, dilution 1;100), BECN1 (#3495, Cell Signaling, USA, dilution 1;100) and BNIP3L/NIX (ab8399, Abcam, UK, dilution 1:100) overnight at 4℃. After incubation with anti-rabbit Alexa Fluor 750-conjugated secondary antibody (ab175733, Abcam, UK, dilution 1:500), the coverslips were mounted in Fluoroshield™ with DABCO (ImmunoBioScience Corp. USA). vWF-FITC (von Willebrand factor-fluorescein; ab8822, Abcam, UK, dilution 1:500, Abcam) was used as an endothelial marker to confirm the endothelial origin of CSF cells^14^. CD41 (GTX113758, GeneTex, USA, dilution 1:100) was used to differentiate endothelial and platelet, and it was confirmed that vWF was an endothelial specific marker in our study. To assess the dysfunctional mitochondria undergoing autophagy in vWF-positive CSF cells, autophagy- or mitophagy-related markers were subjected to co-immunofluorescence stained with MTDR (M22426, Invitrogen, USA, dilution 200 nM), which is widely used as a mitochondria-selective dye to determine mitophagy^35^. Nuclei were counterstained with 4′,6-diamidino-2-phenylindole dihydrochloride (DAPI, D1306, Invitrogen, USA, dilution 300 nM). Fluorescence images were obtained via confocal microscopy at 400 × magnification.

### Real time qRT-PCR

The loss of mitochondrial membrane potential leading to mitochondrial dysfunction results in activation of mitochondrial autophagy. Therefore, autophagy and mitophagy markers such as DAPK1, PINK1, BAX, BNIP3L, and NDP52 were examined in CSF cells^21,22^. Total RNAs were isolated from the CSF cells using TriZOL (Ivitrogen, USA) according to the manufacturer’s instructions. The cDNA was synthesized from 5 μg of RNA using Maxime RT PreMix Kit (iNtRON Biotechnology, Korea). The expression of mitophagy-related genes was measured by qRT-PCR using the 2 × Rotor-Gene SYBR Green PCR Master Mix (Qiagen, Carlsbad, CA, USA) in the Rotor-Gene Q (Qiagen, USA). Primer sequences are presented in the Supplemental Table S1.

### Immunogold staining

We further performed immunogold staining to confirm the location of the most significant differentially expressed markers within the mitochondria of CSF cells in SAH patients with DCI. CSF cells were fixed with 0.1% glutaraldehyde and 2% paraformaldehyde in phosphate buffer at pH 7.4 for 1 h at 4 °C, followed by incubation with post-fixed osmium tetroxide for 30 min at 4 °C. The samples were dehydrated in a graded series of ethanol. The samples were treated with graded ethanol series and embedded in LR white resin (EMS). The sections were then sliced into ultra-thin sections of 80 nm each, and placed on a copper grid. The sections were treated with 0.02 M glycine for 10 min to ensure quench-free aldehyde groups. Sections were then washed with deionized water, blocked in 1% BSA and incubated with rabbit anti-DAPK1 antibodies (PA5-14,044, Invitrogen, USA, dilution 1:100) for 1 h. The grid was washed five times with 0.1% BSA in PBS, and incubated with a secondary antibody conjugated with 10 nm gold (AUR810.011, AURION, Netherlands, dilution 1:100) in 0.1% BSA in PBS. The final samples were stained with uranyl acetate and lead citrate, and then visualized under a transmission electron microscope (JEOL-2100F, USA) at 200 kV.

### Statistical analysis

Continuous variables are expressed as the mean and ± standard deviation (SD). A chi-square or Student’s *t* test was carried out to identify meaningful differences between DCI and non-DCI patients. Comparative analysis via qRT-PCR was performed using the Mann–Whitney U test. The results were presented as the median and 25th–75th percentiles. Quantification of western blots using the relative optical densities with actin protein as the reference and presented as the mean ± standard error of the mean (SEM). Univariate analysis was performed to find relevant factors for the development of DCI. A multivariable logistic regression analysis was then performed to identify independent variables associated with the development of DCI using variables showing *p* value less than 0.20 in the univariate analysis. Statistical analysis was performed with SPSS V.21 (SPSS, Illinois, USA) and GraphPad Prism software (v.6.01; GraphPad Software Inc., San Diego, CA, USA) with a statistical significance indicated at *p* < 0.05.

### Ethical approval

Sample collection and study design were performed according to the principles of the Declaration of Helsinki and were approved by Coordinating Ethnics Committee of the Chuncheon Sacred Heart Hospital.

## Supplementary Information

Supplementary Information.

## Data Availability

Data are available from the corresponding author (JPJ) upon ethical approval from the IRB of the participating hospital.

## References

[1] Lantigua H (2015). Subarachnoid hemorrhage: Who dies, and why?. Crit. Care.

[2] Vergouwen MD (2010). Definition of delayed cerebral ischemia after aneurysmal subarachnoid hemorrhage as an outcome event in clinical trials and observational studies: Proposal of a multidisciplinary research group. Stroke.

[3] Rowland MJ (2012). Delayed cerebral ischaemia after subarachnoid haemorrhage: Looking beyond vasospasm. Br. J. Anaesth..

[4] Ho WM (2018). Autophagy after subarachnoid hemorrhage: Can cell death be good?. Curr. Neuropharmacol..

[5] Lee JY (2009). Activated autophagy pathway in experimental subarachnoid hemorrhage. Brain Res..

[6] Wu W (2015). Pink1-parkin-mediated mitophagy protects mitochondrial integrity and prevents metabolic stress-induced endothelial injury. PLoS ONE.

[7] Hayakawa K (2016). Transfer of mitochondria from astrocytes to neurons after stroke. Nature.

[8] Guan R (2018). Mitophagy, a potential therapeutic target for stroke. J. Biomed. Sci..

[9] Zhang T (2019). Mitophagy reduces oxidative stress via keap1 (kelch-like epichlorohydrin-associated protein 1)/nrf2 (nuclear factor-e2-related factor 2)/phb2 (prohibitin 2) pathway after subarachnoid hemorrhage in rats. Stroke.

[10] Sehba FA (2012). The importance of early brain injury after subarachnoid hemorrhage. Prog. Neurobiol..

[11] Lin, C.L. et al. Cerebral vasospasm after aneurysmal subarachnoid hemorrhage: Mechanism and therapies. *Biomed Res Int*. 679014 (2014). 10.1155/2014/679014 PMC4172888 25276807

[12] Liu F (2018). Mitochondria in ischemic stroke: New insight and implications. Aging Dis..

[13] Galluzzi L (2012). Mitochondria: Master regulators of danger signalling. Nat. Rev. Mol. Cell Biol..

[14] Chou SH (2017). Extracellular mitochondria in cerebrospinal fluid and neurological recovery after subarachnoid hemorrhage. Stroke.

[15] Youn DH (2020). Extracellular mitochondrial dysfunction in cerebrospinal fluid of patients with delayed cerebral ischemia after aneurysmal subarachnoid hemorrhage. Neurocrit. Care.

[16] Baek SH (2014). Modulation of mitochondrial function and autophagy mediates carnosine neuroprotection against ischemic brain damage. Stroke.

[17] Shi RY (2014). Bnip3 interacting with lc3 triggers excessive mitophagy in delayed neuronal death in stroke. CNS Neurosci. Ther..

[18] Chen Y (2017). Reduction in autophagy by (-)-epigallocatechin-3-gallate (egcg): A potential mechanism of prevention of mitochondrial dysfunction after subarachnoid hemorrhage. Mol. Neurobiol..

[19] Cao S (2017). Melatonin-mediated mitophagy protects against early brain injury after subarachnoid hemorrhage through inhibition of nlrp3 inflammasome activation. Sci. Rep..

[20] Leclerc JL (2015). Haptoglobin phenotype predicts the development of focal and global cerebral vasospasm and may influence outcomes after aneurysmal subarachnoid hemorrhage. Proc. Natl. Acad. Sci. U S A.

[21] Shang T, Joseph J, Hillard CJ, Kalyanaraman B (2005). Death-associated protein kinase as a sensor of mitochondrial membrane potential: Role of lysosome in mitochondrial toxin-induced cell death. J. Biol. Chem..

[22] Wang Y, Liu N, Lu B (2019). Mechanisms and roles of mitophagy in neurodegenerative diseases. CNS Neurosci. Ther..

[23] Wang S (2017). Dapk1 signaling pathways in stroke: From mechanisms to therapies. Mol. Neurobiol..

[24] Tu W (2010). Dapk1 interaction with nmda receptor nr2b subunits mediates brain damage in stroke. Cell.

[25] Lai MZ, Chen RH (2014). Regulation of inflammation by dapk. Apoptosis.

[26] Chuang YT (2008). The tumor suppressor death-associated protein kinase targets to tcr-stimulated nf-kappa b activation. J. Immunol..

[27] Masaki T, Sawamura T (2006). Endothelin and endothelial dysfunction. Proc. Jpn. Acad. Ser. B Phys. Biol. Sci..

[28] Lackner P (2010). Cellular microparticles as a marker for cerebral vasospasm in spontaneous subarachnoid hemorrhage. Stroke.

[29] Zhang J (2013). Autophagy and mitophagy in cellular damage control. Redox Biol..

[30] Park JJ (2020). A preliminary study of the association between sox17 gene variants and intracranial aneurysms using exome sequencing. J. Korean Neurosurg. Soc..

[31] Seto S, Tsujimura K, Horii T, Koide Y (2013). Autophagy adaptor protein p62/sqstm1 and autophagy-related gene atg5 mediate autophagosome formation in response to mycobacterium tuberculosis infection in dendritic cells. PLoS ONE.

[32] Comincini S (2017). Identification of autophagy-related genes and their regulatory mirnas associated with celiac disease in children. Int. J. Mol. Sci..

[33] Okazaki T, Kuroda Y (2018). Aneurysmal subarachnoid hemorrhage: Intensive care for improving neurological outcome. J. Intensive Care..

[34] Samagh N, Bhagat H, Jangra K (2019). Monitoring cerebral vasospasm: How much can we rely on transcranial doppler. J. Anaesthesiol. Clin. Pharmacol..

[35] Mauro-Lizcano M (2015). New method to assess mitophagy flux by flow cytometry. Autophagy.

