# Supplementary material for: Mitochondrial dysfunction associated with autophagy and mitophagy in cerebrospinal fluid cells of patients with delayed cerebral ischemia following subarachnoid hemorrhage
Source: Sci Rep. 2021 Aug 13;11:16512. doi: 10.1038/s41598-021-96092-2 (PMC8363614; doi:10.1038/s41598-021-96092-2)

**Supplemental Data**

**Protocol for DCI diagnosis and inter-assessor agreement**

1^st^ Reviewer: Jong Kook Rhim, neurointerventionist with more than 10 years of experience

2^nd^ Reviewer: Heung Cheol Kim, neuroradiologist with more than 20 years of experience

3^rd^ Reviewer: Jin Pyeong Jeon, neurointerventionist with more than six years of experience

Each reviewer reviewed the medical records and radiological findings independently, followed by a comparison of their DCI diagnosis. Next, JP Jeon reported the results based on inter-assessor reliability with Cohen’s kappa index. All disagreements were resolved by JP Jeon. Cohen’s kappa was 0.889, indicating almost perfect agreement.

| n = 56 | | Kim’s decision | |
| --- | --- | --- | --- |
|  |  | Non-DCI | DCI |
| Rhim’s decision | Non-DCI | 32 | 1 |
|  | DCI | 2 | 21 |

**Supplemental Table S1.** Primer sequences used in this study

| **Names** | **Primer sequence (5’→3’)** | **Product size (bp)** | **Position** |
| --- | --- | --- | --- |
| ***qPCR primers**** | | | |
| DAPK1-F | GACCGTGAAGCATTACCTGAG | 124 | 3856-3980 |
| DAPK1-R | GCTGCTGAAGCTTTCCTTGTA |  |  |
| BNIP3L-F | ACAACAACAACTGC GAGGAAA | 144 | 121-265 |
| BNIP3L-R | GAGGATGAGGATGGTACGTGT |  |  |
| BAX-F | GTTTCATCCAGGATCGAGCAG | 145 | 5827-5972 |
| BAX-R | CTGCAGCTCCATGTTACTGTC |  |  |
| PINK1-F | GTATGAAGCCACCATGCCTAC | 153 | 604-757 |
| PINK1-R | CATCATCTTGATGGCCAAGGGTC |  |  |
| ULK-F | AACAAGAAGAACCTCG CCAAG | 145 | 413-558 |
| ULK-R | CCGTTGCAGTACTCCATAACC |  |  |
| NDP52-F | CCCCTACCATGGAGGAGACC | 260 47-307 | |
| NDP52-R | CCTGCAATTTCTGTCCTTGGC |  |  |
| Actin-F | CATGTACGTTGCTATCCAGGC | 249 | 477-726 |
| Actin-R | CTCCTTAATGTC ACGCACGA |  |  |

*Primer positions of qRT-PCR indicate the base from the start codon. The first nucleotide of the start codon is defined as position 1.

**Supplemental Table S2.** The Comparison of the expression of autophagy and mitophagy markers via western blot analysis between DCI and non-DCI patients. *, **, and *** represent p-value of less than of <0.05, 0.01, and 0.001, respectively.

| Variables | Non-DCI (n=6) | | DCI (n=6) | | Statistical |
| --- | --- | --- | --- | --- | --- |
|  | Mean | SEM | Mean | SEM | Significance |
| pBeclin1 | 0.203 | 0.023 | 1.660 | 0.183 | *** |
| Beclin1 | 0.131 | 0.008 | 0.651 | 0.137 | *** |
| BNIP3L | 0.034 | 0.016 | 0.585 | 0.079 | *** |
| DAPK1 | 0.179 | 0.032 | 0.912 | 0.170 | *** |
| PINK1 | 0.496 | 0.050 | 1.267 | 0.159 | * |
| LC3 | 0.220 | 0.095 | 1.089 | 0.301 | *** |
| P62 | 0.285 | 0.039 | 0.073 | 0.004 | *** |

**Supplemental Table S3**. Comparison of autophagy and mitophagy markers expressed in CSF cells of SAH patients according to the initial clinical severity of high Hunt and Hess (H-H) grades IV and V and non-high H-H grades I, II, and III.

| Variables | Non-high H-H grade  (n=34) | High H-H grade  (n=22) | p-value |
| --- | --- | --- | --- |
| DAPK1 | 0.0784 (0.0234 – 0.1920) | 0.1780 (0.0576 – 0.2810) | 0.166 |
| BNIP3L | 0.0563 (0.0254 – 0.1110) | 0.0901 (0.0308 – 0.1990) | 0.347 |
| PINK1 | 0.0548 (0.0368 – 0.0732) | 0.0432 (0.0181 – 0.0769) | 0.591 |
| BAX | 0.1730 (0.0007 – 0.0129) | 0.2270 (0.1350 – 0.3240) | 0.056 |
| ULK1 | 0.0033 (0.0234 – 0.1920) | 0.0073 (0.0037 – 0.0195) | 0.085 |
| NDP52 | 0.1810 (0.0532 – 0.2750) | 0.1780 (0.0430 – 0.2620) | 0.788 |

**Supplemental Table S4**. Comparison of autophagy and mitophagy markers expressed in CSF cells of SAH patients according to clinical outcomes 3 months after ictus.

| Variables | Good outcome  (n=36) | Poor outcome  (n=20) | p-value |
| --- | --- | --- | --- |
| DAPK1 | 0.0967 (0.0239 – 0.1980) | 0.1160 (0.0353 – 0.2660) | 0.831 |
| BNIP3L | 0.0629 (0.0341 – 0.1380) | 0.0539 (0.0201 – 0.1660) | 0.555 |
| PINK1 | 0.0521 (0.0364 – 0.0720) | 0.0432 (0.0127 – 0.0795) | 0.505 |
| BAX | 0.1800 (0.0992 – 0.2170) | 0.2080 (0.1230 – 0.3910) | 0.235 |
| ULK1 | 0.0038 (0.0006 – 0.0117) | 0.0068 (0.0035 – 0.0305) | 0.055 |
| NDP52 | 0.1970 (0.0494 – 0.2790) | 0.1040 (0.0430 – 0.2430) | 0.330 |

**Supplemental Figure S1.** Full scans of original unprocessed western blots presented in Figure 2B. In red, the cropped area used in the main figure.


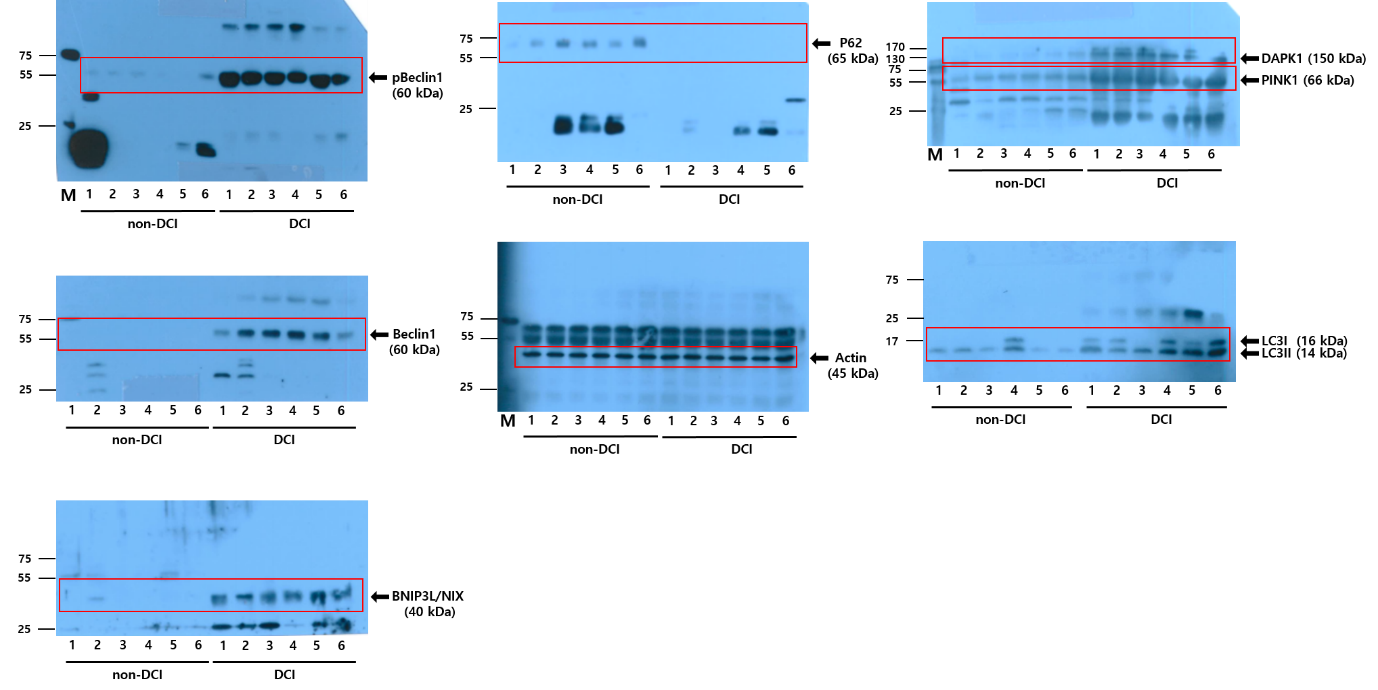

Supplement: Supplementary file 1 — Supplementary Information. [file 41598_2021_96092_MOESM1_ESM.docx]
